# Evaluation of IGFBP-7 DNA methylation changes and serum protein variation in Swedish subjects with and without type 2 diabetes

**DOI:** 10.1186/1868-7083-5-20

**Published:** 2013-11-04

**Authors:** Harvest F Gu, Tianwei Gu, Agneta Hilding, Yiming Zhu, Lars Kärvestedt, Claes-Göran Östenson, Maode Lai, Masahiko Kutsukake, Jan Frystyk, Kazuhiro Tamura, Kerstin Brismar

**Affiliations:** 1 Rolf Luft Research Center for Diabetes and Endocrinology, Department of Molecular Medicine and Surgery, Karolinska Institutet, Karolinska University Hospital, Stockholm SE-171 76, Sweden; 2 Department of Molecular Pathology, School of Medicine, Zhejiang University, Hangzhou, PR China; 3 Department of Endocrine Pharmacology, Tokyo University of Pharmacy and Life Sciences, Tokyo, Japan; 4 Medical Research Laboratory, Department of Clinical Medicine, Faculty of Health, Aarhus University, Aarhus, Denmark; 5 Department of Endocrinology and Internal Medicine, Aarhus University Hospital, Aarhus, Denmark

**Keywords:** IGF-1, IGFBP-1, IGFBP-7, Insulin, Type 2 diabetes

## Abstract

**Background:**

Insulin-like growth factor-binding protein 7 (IGFBP-7) is able to interact with insulin-like growth factor 1 (IGF-1) as well as insulin. Previous studies have suggested that serum IGFBP-7 levels may be associated with insulin resistance in type 2 diabetes (T2D). This study aimed to evaluate IGFBP-7 serum protein and *IGFBP7* DNA methylation levels in the subjects with and without T2D.

**Results:**

A total of 340 Swedish subjects including 100 newly diagnosed T2D patients (50 women/50 men), 100 age-matched nondiabetic control subjects (50/50) and 140 treated T2D patients (54/86) were studied. Serum IGFBP-7 levels were measured with a novel ELISA. IGF1, IGFBP-1, and insulin were determined by in-house radioimmunoassays. DNA methylation levels in the *IGFBP7* gene were analyzed with a bisulfite-pyrosequencing technique. Serum IGFBP-7 protein levels were similar among nondiabetic subjects, newly diagnosed, and treated T2D patients and were not correlated with *IGFBP7* DNA methylation. However, *IGFBP7* DNA methylation was increased in men with newly diagnosed T2D compared with nondiabetic controls (17.6% vs. 12.5%, *P* < 0.01). Serum IGFBP-7 levels correlated (*r* = 0.331, *P* = 0.019) with serum IGFBP-1 levels, a marker of insulin production, in men but not women with newly diagnosed T2D.

**Conclusions:**

This study demonstrates for the first time that *IGFBP7* DNA methylation levels are increased in Swedish men with newly diagnosed T2D. The correlation between IGFBP-7 and IGFBP-1 suggests that low IGFBP-7 may be associated with insulin resistance in T2D.

## Background

Insulin-like growth factor 1 (IGF-1) is a polypeptide hormone, which shares structural homology and downstream signaling pathways with insulin. Several studies have demonstrated that IGF-1 has insulin-like effects, stimulating peripheral uptake of glucose and free fatty acids [1-3]. Low levels of serum IGF-1 are associated with an increasing risk of developing type 2 diabetes (T2D) and obesity [4,5]. IGF-1 circulates in blood predominantly complexed to specific IGF-binding proteins (IGFBP-1). There are six binding proteins (IGFBP-1 to 6) exhibiting high affinity to IGF-1 [1-3]. IGFBP-1 has been shown to prolong the half-life of IGF-1 and IGF-2. Elevations in insulin suppress the hepatic production of IGFBP-1, resulting in decreased circulating levels. Circulating IGFBP-1 levels are inversely correlated with 24-hour insulin secretion, insulin sensitivity, and an increased risk of cardiovascular diseases [6-9]. Finally, low IGFBP-1 levels may be permissive for the development of metabolic diseases, such as T2D, obesity, and related cardiovascular complications [10-13].

IGFBP-7 (also known as IGFBP-rP1, MAC25, PSF, TAF, FSTL2, or PGI2-stimulating factor) is an additional member of the IGFBP family. Unlike the high affinity of the other six IGFBPs, IGFBP-7 exhibits low affinity for IGF-1, but has a relatively high affinity for insulin [14,15]. It is hypothesized that IGFBP-7 may interfere with insulin action and subsequently play a role in the development of diabetes and diabetic vascular complication. There are three reports evaluating IGFBP-7 in T2D. In 2006, Lopez-Bermejo *et al*. [16] measured serum IGFBP-7 levels in 43 men with T2D and 113 nondiabetic subjects and found increased serum IGFBP-7 levels to be associated with insulin resistance. One year later, the same research group [17] detected serum IGFBP-7 levels in 24 male T2D patients with high-ferritin and suggested that vascular function was linked to serum IGFBP-7 levels in these patients. In 2008, Kutsukake *et al*. [18] developed a novel IGFBP-7 ELISA using two IGFBP-7 antibodies and analyzed serum IGFBP-7 levels in 33 male hemodialysis patients, including 18 patients with and 15 patients without T2D. Their data indicated that hemodialysis patients with T2D had higher serum IGFBP-7 levels than the hemodialysis patients without T2D.

These three studies suggested a possible association between IGFBP-7 and T2D. However, the sample sizes of T2D patients in the studies were relatively small and no female patient with T2D was included. Furthermore, recent studies have shown that epigenetic changes provide a link for the translation of environmental exposures into susceptibility for T2D. Epigenetic studies may provide useful information for a better understanding of the pathogenesis of T2D [19-21]. To our knowledge, no epigenetic study of the *IGFBP7* gene in T2D has been reported. In this study, we have determined serum IGFBP-7 levels in a cohort consisting of 340 Swedish men and women including newly diagnosed T2D patients, age-matched nondiabetic controls and anti-diabetic treated T2D patients. In addition, we analyzed levels of *IGFBP7* DNA methylation. Our aims were to evaluate serum protein and DNA methylation levels of IGFBP-7 in T2D.

## Results

### Serum IGFBP-7 levels and correlation with IGFBP-1

Serum IGFBP-7 protein levels in both men and women with normal glucose tolerance (NGT), newly diagnosed T2D, and anti-diabetic treated T2D are presented in Table 1. No statistical significant difference in serum IGFBP-7 levels was found between the three groups, neither in analyses when data for men and women were combined nor in sex-specific analyses.

**Table 1:** Clinical and laboratory variables in Swedish subjects with normal glucose tolerance and in the patients with type 2 diabetes

		**Stockholm Diabetes Prevention Program**		**Kronan**
		**Normal glucose tolerance**	**Newly diagnosed type 2 diabetes**	**Type 2 diabetes**
*N* = All (women/men)		100 (50/50)	100 (50/50)	140 (54/86)
Age (years)	All	58 (57 to 58)	58 (57 to 58)	61 (60 to 63)^†††^^§§§^
	Women	57 (55 to 58)	57 (55 to 58)	62 (60 to 64)^†††§§§^
	Men	58 (57 to 59)	58 (57 to 59)	62 (60 to 63)^††§§^
Body mass index (kg/m^2^)	All	24.4 (23.8 to 25.1)	30.7 (29.4 to 31.9)^†††^	29.3 (28.6 to 30.1)^†††^
	Women	23.4 (22.7 to 24.1)	32.2 (30.2 to 34.3)^†††^	29.7 (28.4 to 30.9)^†††§^
	Men	25.4 (24.4 to 26.4)***	29.1 (27.8 to 30.4)^†††^*	29.1 (28.1 to 30.2)^†††^
Waist and hip ratio	All	0.86 (0.84 to 0.87)	0.93 (0.92 to 0.94)^†††^	0.95 (0.94 to 0.96)^†††§^
	Women	0.82 (0.80 to 0.83)	0.90 (0.88 to 0.91)^†††^	0.90 (0.89 to 0.92)^†††^
	Men	0.90 (0.88 to 0.91)***	0.96 (0.95 to 0.98)^†††^***	0.98 (0.97 to 0.99)^†††^***
Systolic blood pressure (mm Hg)	All	130 (127 to 134)	146 (143 to 150)^†††^	147 (144 to 150)^†††^
	Women	129 (124 to 133)	147 (141 to 152)^†††^	149 (145 to 154)^†††^
	Men	132 (126 to 137)	146 (141 to 150)^†††^	146 (142 to 150)^†††^
Diastolic blood pressure (mm Hg)	All	80 (78 to 82)	87 (85 to 90)^†††^	83 (81 to 84)^§§§^
	Women	79 (77 to 82)	86 (83 to 89)^††^	81 (78 to 83)^§^
	Men	82 (79 to 84)	89 (86 to 92)^†††^	84 (82 to 86)^§^
Fasting glucose (mmol/l)	All	4.8 (4.7 to 4.9)	6.8 (6.4 to 7.1)^†††^	9.0 (8.5 to 9.5)^†††§§§^
	Women	4.6 (4.4 to 4.7)	6.6 (6.2 to 6.9)^†††^	9.2 (8.4 to 9.9)^†††§§§^
	Men	5.0 (4.9 to 5.1)***	6.9 (6.4 to 7.5)^†††^	8.9 (8.2 to 9.5)^†††§§§^
Fasting insulin (pmol/l)^a^	All	73 (68 to 78)	140 (126 to 156)^†††^	135 (124 to 147)^†††^
	Women	70 (64 to 76)	150 (130 to 173)^†††^	138 (123 to 154)^†††^
	Men	77 (69 to 85)	131 (112 to 154)^†††^	133 (118 to 150)^†††^
Homeostasis model of assessment: insulin resistance^a^	All	2.56 (2.37 to 2.78)	6.86 (6.03 to 7.79)	8.53 (7.66 to 9.50)^†††§^
	Women	2.33 (2.11 to 2.59)	7.20 (6.09 to 8.52)	9.00 (7.78 to 10.41)^†††^
	Men	2.82 (2.49 to 3.18)*	6.52 (5.35 to 7.96)	8.25 (7.09 to 9.60)^†††^
IGF-I (μg/l)^a^	All	156 (147 to 167)	154 (144 to 165)	117 (107 to 128)^†††§§§^
	Women	154 (141 to 167)	157 (143 to 172)	122 (106 to 141)^†§§^
	Men	159 (145 to 175)	152 (138 to 167)	114 (102 to 128)^†††§§§^
IGFBP-1 (μg/l)^a^	All	42 (38 to 47)	23 (20 to 26)^†††^	17 (14 to 20)^†††§§^
	Women	57 (50 to 65)	28 (24 to 33)^†††^	18 (14 to 22)^†††§§^
	Men	31 (27 to 37)***	19 (16 to 23)^††^**	16 (13 to 20)^†††^
IGFBP-7 (μg/l)^a^	All	20.5 (18.7 to 22.3)	19.6 (17.8 to 21.5)	19.2 (17.9 to 20.6)
	Women	19.0 (16.8 to 21.4)	20.0 (17.0 to 23.5)	18.8 (16.7 to 21.7)
	Men	22.1 (19.4 to 25.1)	19.2 (17.3 to 21.3)	19.5 (17.8 to 21.3)

Data were expressed as means (95% confidence interval (CI)) for normally distributed variables and as geometric means (95% CI) for ^a^non-normally distributed variables; Comparison between the three groups was performed by one-way analysis of variance (ANOVA), and if significant followed by Tukey’s post-hoc test; ^†^*P* < 0.05, ^††^*P* < 0.01, ^†††^*P* < 0.001 vs. Stockholm Diabetes Prevention Program NGT and ^§^*P* < 0.05, ^§§^*P* < 0.01, ^§§§^*P* < 0.001 vs. Stockholm Diabetes Prevention Program type 2 diabetes; Comparison of men and women within groups was performed by unpaired *t* test; *P < 0.05, **P < 0.01, ***P < 0.001.

**Figure 1:**
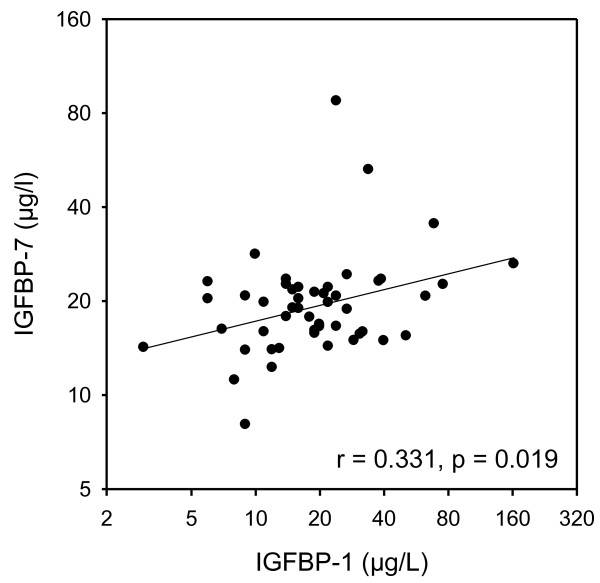
**Relationship between serum IGFBP-7 and IGFBP-1 in Swedish men with newly diagnosed type 2 diabetes.** T2D, type 2 diabetes.

We then analyzed the correlations between serum IGFBP-7 protein levels and other laboratory variables, including fasting glucose, fasting insulin, homeostasis model of assessment insulin resistance (HOMA-IR), IGF-1, and IGFBP-1. In patients with newly diagnosed T2D, there was no correlation between IGFBP-7 and fasting glucose, fasting insulin, or HOMA-IR. However, a tendency towards a significant negative correlation was found between IGFBP-7 and IGF-1 (*r* = -0.258, *P* = 0.070) in men with newly diagnosed T2D (Table 2). Furthermore, IGFBP-7 correlated positively with IGFBP-1 in these male patients (*r* = 0.331, *P* = 0.019, Figure 1). Similar correlations were not observed in newly diagnosed women with newly diagnosed T2D. Apart from these correlations, none was observed.

**Table 2:** Univariate correlations between IGFBP-7 and other laboratory variables in Swedish subjects with normal glucose tolerance and newly diagnosed type 2 diabetes

	**Normal glucose tolerance**			**Newly diagnosed type 2 diabetes**		
	**All**	**Women**	**Men**	**All**	**Women**	**Men**
	***n *** **= 100**	***n *** **= 50**	***n *** **= 50**	***n *** **= 100**	***n *** **= 50**	***n = *** **50**
Fasting glucose	*r* = 0.016	*r* = -0.158	*r* = 0.042	*r* = 0.016	*r* = -0.158	*r* = 0.042
	*P* = 0.877	*P* = 0.273	*P* = 0.771	*P* = 0.877	*P* = 0.273	*P* = 0.771
Fasting insulin ^a^	*r* = 0.080	*r* = 0.115	*r* = 0.013	*r* = 0.039	*r* = 0.136	*r* = -0.107
	*P* = 0.432	*P* = 0.427	*P* = 0.929	*P* = 0.702	*P* = 0.348	*P* = 0.462
HOMA-IR ^a^	*r* = 0.068	*r* = 0.047	*r* = 0.016	*r* = 0.012	*r* = 0.137	*r* = - 0.158
	*P* = 0.499	*P* = 0.745	*P* = 0.914	*P* = 0.904	*P* = 0.344	*P* = 0.273
IGF-I ^a^	*r* = 0.053	*r* = 0.102	*r* = -0.004	*r* = -0.144	*r =* -0.080	*r* = -0.258
	*P* = 0.599	*P* = 0.480	*P* = 0.977	*P =* 0.153	*P* = 0.583	*P* = 0.070
IGFBP-1 ^a^	*r* = 0.003	*r* = 0.149	*r* = 0.075	*r* = 0.114	*r* = -0.071	*r* = 0.331
	*P =* 0.978	*P* = 0.301	*P* = 0.607	*P* = 0.261	*P* = 0.626	*P* = 0.019

^a^Log-transformed before analyses.

### DNA methylation levels of the IGFBP7 gene

**Figure 2:**
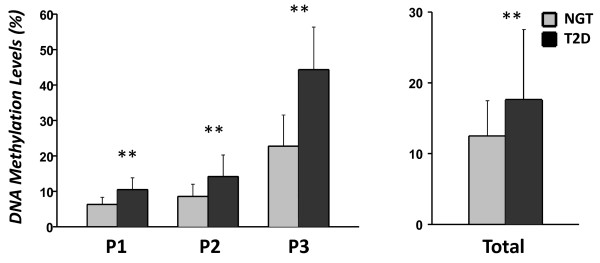
***IGFBP7 *DNA methylation levels in Swedish men with normal glucose tolerance and newly diagnosed type 2 diabetes.** Data were mean ± standard deviation. NGT, normal glucose tolerance; T2D, type 2 diabetes; **P < 0.01.

To further investigate whether IGFBP-7 plays a role in T2D, we analyzed DNA methylation of the *IGFBP7* gene in men with NGT or newly diagnosed T2D, respectively. Figure 2 demonstrates that *IGFBP7* DNA methylation levels were significantly increased in newly diagnosed T2D patients compared with NGT subjects at all three CpG sites (P1: 10.4% vs. 6.2%; P2: 14.3% vs. 8.6% and P3: 44.4% vs. 22.7%, all *P* < 0.01). Combined data from all three CpG sites showed that genomic DNA methylation levels of the *IGFBP7* gene in T2D were increased compared with subjects with NGT (17.6% vs. 12.5%, *P* < 0.01).

## Discussion

We have determined serum IGFBP-7 levels in newly diagnosed T2D patients, age-matched nondiabetic controls and anti-diabetic treated T2D patients and for the first time found a correlation between IGFBP-7 and IGFBP-1 in men with newly diagnosed T2D. Moreover, we analyzed the levels of *IGFBP7* DNA methylation and found that *IGFBP7* DNA methylation levels were higher in male T2D patients than in nondiabetic subjects.

IGFBP-7 is widely expressed in various tissues, including the brain, lung, prostate, and gastrointestinal tract and the protein is also detectable in the circulation [14,15]. In this study, we measured serum IGFBP-7 protein levels using a novel sandwich ELISA protocol [18]. Data demonstrated that serum IGFBP-7 protein mean concentrations were 19 to 22 μg/l in Swedish men with and without T2D and 18.8 to 20.0 μg/l in women. Further, no statistically significant difference in serum IGFBP-7 protein levels was found between the subjects with NGT and newly diagnosed T2D or anti-diabetic treated T2D patients. Previously, Lopez-Bermejo *et al*. [16] reported that the mean levels of serum IGFBP-7 in Spanish men without and with T2D were 26 to 28 μg/l using an enzyme-linked immune-sorbent assay. Data in their study implicated that IGFBP-7 was associated with insulin resistance [16]. However, the association of IGFBP-7 with insulin resistance (determined as HOMA-IR) was not observed in our study. The difference in results may be due to sample size and the selection criteria of the T2D patients. In this study, the sample size was larger and the patients were newly diagnosed, as compared with previous reports [16-18].

Low serum IGFBP-1 levels are found to be associated with hyperinsulinemia, insulin sensitivity, development of T2D, and obesity [10-12]. In this study, low serum IGFBP-7 levels correlated with low serum IGFBP-1 levels in men with newly diagnosed T2D. We acknowledge that this observation may not be causal. Nevertheless, we have a hypothesis that low IGFBP-7 levels may be related to increased insulin and consequently associated to insulin resistance. However, further mechanistic investigations are needed to test this hypothesis.

Several studies have demonstrated that increased DNA methylation levels of the *IGFBP7* gene are associated with colorectal cancers and lung and prostate cancer. IGFBP-7 may play a tumor-suppressor role in carcinogenesis of these cancers, mainly through inactivation of its DNA methylation [22-25]. DNA methylation is involved in the regulation and expression of genes [26]. This epigenetic mechanism is believed to be a contributing factor to pathological conditions, such as T2D [19-21]. In this study, we have, for the first time, provided evidence that DNA methylation levels in the *IGFBP7* gene are increased in Swedish men with newly diagnosed T2D. This finding implicates that *IGFBP7* DNA methylation may be associated with early diagnosis of T2D. Generally, DNA methylation of cytosine residues in CpG di-nucleotides may lead to transcriptional silencing of the associated gene. In this study, we did not find any correlation between serum IGFBP-7 protein levels and *IGFBP7* DNA methylation. One explanation is that DNA methylation may reduce gene transcription but not translation [26]. Furthermore, we have analyzed *IGFBP7* DNA methylation levels in blood cells. However, we believe that investigations of tissue-specific *IGFBP7* DNA methylation in T2D patients or diabetic animal models are necessary to better understand the correlation of *IGFBP7* DNA methylation with serum IGFBP-7 protein levels.

Clinical observations in many populations, including Swedish cohorts, have shown that the prevalence and subsequent incidence of T2D are higher in men than in women [27,28]. The endogenous sex hormones may differentially modulate glycemic status and risk of T2D in men and women [29]. In this study, we found that *IGFBP7* DNA methylation levels were associated with T2D in men but not in women. We do not know the mechanism of the sex-based difference in methylation *IGFBP7*, but information from public genome databases implicates that the *IGFBP7* mRNA expression levels are high in the prostate and testes but low in the breast and ovaries [30]. Furthermore, IGFBP-7 is known to be secreted by adipose tissues and, indeed, a sex-linked difference is seen in hormones secreted by adipocytes, such as adiponectin and leptin [31,32].

## Conclusions

This study demonstrates for the first time that *IGFBP7* DNA methylation is increased in Swedish men with newly diagnosed T2D compared with subjects with NGT. Low serum IGFBP-7 levels are related to low IGFBP-1 and subsequently associated with insulin resistance in T2D.

## Methods

### Subjects

A total of 340 subjects were selected from the Stockholm Diabetes Prevention Program (SDPP) and the Kronan study. All subjects were Swedish. In SDPP, men and women aged 35 to 56 years from five municipalities in the region of Stockholm, Sweden, were included in an epidemiological survey [33]. A follow-up study was performed after 10 and 8 years, respectively, in the SDPP and Kronan studies. Both baseline and follow-up studies consisted of a questionnaire covering lifestyle factors, a health examination and an oral glucose tolerance test. Based upon this test, all participants were categorized into glucose tolerance groups according to World Health Organization criteria [34]. For this study, 100 newly diagnosed T2D patients at the follow-up study, having either NGT or impaired glucose tolerance at baseline (50 women, 50 men), were selected. These T2D patients received no anti-diabetic medication when the blood samples were taken and the phenotypes recorded. One hundred nondiabetic controls (50 women, 50 men) were selected among those having NGT at both baseline and follow-up and matched to newly diagnosed T2D patients by sex and age. In the Kronan study, 140 patients with T2D (54 women, 86 men) were selected from three health care centers, Kronan, Hallonbergen, and Rissne, within the municipality of Sundbyberg, Stockholm, Sweden [35]. Patients with latent autoimmune diabetes of adults were excluded in this study. All patients received anti-diabetes treatment: 24% were treated with diet alone, 46% with oral hypoglycemic agents, 22% with insulin and 8% with a combination of insulin and oral hypoglycemic agents.

Informed consent from all participants was received. The study was approved by the Ethics Committee of Karolinska University Hospital, and was performed in accordance with the Declaration of Helsinki.

### DNA extraction and bisulfite treatment

Genomic DNA was extracted from peripheral blood using the Gentra Puregene Blood Kit (Qiagen, Hilden, Germany). DNA samples were stored at -80°C until use. For epigenetic analysis, bisulfite treatment of extracted DNA samples was done with the EpiTect Bisulfite Kit (Qiagen). This kit enables complete conversion of unmethylated cytosine to uracil and subsequent purification in less than 6 hours. This highly sensitive method utilizes innovative protection against DNA degradation and ensures high conversion rates of over 99%. The experiments for DNA extraction and bisulfite treatment were conducted according to the manufacturer instruction.

### IGFBP7 methylation analyses

The *IGFBP7* gene is located in chromosome 4q12. In the *IGFBP7* gene, there are three CpG sites (**C**GCT**C**GTGCCCACCTTGCT**C**GT, NT_022853.15) as indicated with the bold letter **C** and recorded as P1 to P3 (P for position). In a CpG site sequence, 5-methyl cytosine is followed by guanosine, which is the dominating type of methylation pattern in mammals. PyroMark CpG assay for the *IGFBP7* gene methylation analysis (the detailed sequence information is recorded at ID: PM00112903, Qiagen) and PyroMark PCR kit (Qiagen) were used. Methylation levels of these CpG sites were detected using the PyroMark Gold 96 Reagent Kit (Qiagen) and the PyroMark Q96 ID Pyrosequencing System (Biotage, Uppsala, Sweden). Pyrosequencing methylation analysis of CpG sites is sensitive and accurate [36,37]. PyroQ-CpG software (Biotage) was used for methylation data analysis. Unmethylated bisulfite converted and unconverted DNA samples (Qiagen) were used for control of conversion efficiency of the bisulfite treatment and accuracy in methylation analyses.

### IGFBP-7 measurement

IGFBP-7 levels in serum were measured using a novel sandwich ELISA, which incorporated a polyclonal and a monoclonal anti-IGFBP-7 antibody (R&D Systems Inc., Abingdon, UK) as previously described [18]. Interassay and intra-assay coefficients of variation were 18.7% and 11.2%, respectively. The average recovery rate was approximately 100%. The sensitivity of the assay was 0.3 μg/ml.

### Glucose, insulin, IGF-1, and IGFBP-1 measurements

Fasting plasma glucose levels were analyzed in duplicate with a glucose oxidase analyzer (Yellow Springs, OH, USA). Fasting serum insulin levels were assayed by radioimmunoassay using our own antibodies, human insulin as a standard, and charcoal addition to separate antibody-bound and free insulin [38]. The same serum samples for measuring IGFBP-7 were used for measurement of IGF-1 and IGFBP-1. Measurement of IGF-1 in serum was performed after acid ethanol extraction and cryoprecipitation. Serum IGF-1 levels were determined by radioimmunoassay using des [1-3] IGF-1 as tracer. Intra-assay and interassay coefficients of variation were 4% and 11%, respectively. Serum IGFBP1 levels were measured by an in-house radioimmunoassay with intra-assay and interassay coefficients of variation of 3 and 10%, respectively. The assay protocols have been described previously [39,40].

### Statistical analysis

Data are presented as means ± standard deviation or as means with 95% CI for normally distributed variables. Non-normally distributed variables were log-transformed before analysis to normalize their distribution; their data are given as geometric means with 95% CI. Comparisons between independent groups were performed by unpaired *t* test or by one-way ANOVA, followed by Tukey’s post-hoc test. Linear regression analysis was used to examine the relationship between variables. Homeostasis model of assessment was used to assess insulin resistance index (HOMA-IR) and calculated as:

(fasting serum glucose mmol/l × fasting serum insulin mU/l)/22.5.

All statistical analyses were performed using Statistica, version 11 (StatSoft, Tulsa, OK, USA). A *P* value of <0.05 was considered statistically significant.

## Abbreviations

ANOVA: Analysis of variance; CI: Confidence interval; ELISA: Enzyme-linked immunosorbent assay; HOMA: Homeostasis model of assessment; IGF-1: Insulin-like growth factor 1; IGFBP-1: Insulin-like growth factor-binding protein 1; IGFBP-7: Insulin-like growth factor-binding protein 7; IR: Insulin resistance; NGT: Normal glucose tolerance; PCR: Polymerase chain reaction; SDPP: Stockholm Diabetes Prevention Program; T2D: Type 2 diabetes.

## Competing interest

The authors declare that they have no competing interests.

## Authors’ contributions

HFG and KB designed the study; TG, HFG, MK, KT, YZ, ML, AH, LK, CGÖ, and JF collected experimental and clinical data; AH, TG, and HFG analyzed the data; HFG and KB wrote the manuscript. All authors contributed to data interpretation, discussion, and revision of the manuscript. All authors read and approved the final manuscript.
